# Design of a prostate cancer patient navigation intervention for a Veterans Affairs hospital

**DOI:** 10.1186/1472-6963-12-340

**Published:** 2012-09-25

**Authors:** Narissa J Nonzee, June M McKoy, Alfred W Rademaker, Peter Byer, Thanh Ha Luu, Dachao Liu, Elizabeth A Richey, Athena T Samaras, Genna Panucci, XinQi Dong, Melissa A Simon

**Affiliations:** 1 Robert H. Lurie Comprehensive Cancer Center of Northwestern University, Chicago, IL, USA; 2 Jesse Brown VA Medical Center, Chicago, IL, USA; 3 Department of Medicine, Division of General Internal Medicine & Geriatrics, Northwestern University Feinberg School of Medicine, Chicago, IL, USA; 4 Department of Preventive Medicine, Northwestern University Feinberg School of Medicine, Chicago, IL, USA; 5 Department of Obstetrics and Gynecology, Northwestern University Feinberg School of Medicine, Chicago, IL, 60611, USA; 6 Dartmouth College, Geisel School of Medicine, Hanover, NH, USA; 7 Harvard School of Public Health, Boston, MA, USA; 8 Rush University Institute for Healthy Aging, Chicago, IL, USA

**Keywords:** Patient navigation, Prostate cancer, Cancer health disparities, Veterans

## Abstract

**Background:**

Patient navigation programs have been launched nationwide in an attempt to reduce racial/ethnic and socio-demographic disparities in cancer care, but few have evaluated outcomes in the prostate cancer setting. The National Cancer Institute-funded Chicago Patient Navigation Research Program (C-PNRP) aims to implement and evaluate the efficacy of a patient navigation intervention for predominantly low-income minority patients with an abnormal prostate cancer screening test at a Veterans Affairs (VA) hospital in Chicago.

**Methods/Design:**

From 2006 through 2010, C-PNRP implemented a quasi-experimental intervention whereby trained social worker and lay health navigators worked with veterans with an abnormal prostate screen to proactively identify and resolve personal and systems barriers to care. Men were enrolled at a VA urology clinic and were selected to receive navigated versus usual care based on clinic day. Patient navigators performed activities to facilitate timely follow-up such as appointment reminders, transportation coordination, cancer education, scheduling assistance, and social support as needed. Primary outcome measures included time (days) from abnormal screening to diagnosis and time from diagnosis to treatment initiation. Secondary outcomes included psychosocial and demographic predictors of non-compliance and patient satisfaction. Dates of screening, follow-up visits, and treatment were obtained through chart audit, and questionnaires were administered at baseline, after diagnosis, and after treatment initiation. At the VA, 546 patients were enrolled in the study (245 in the navigated arm, 245 in the records-based control arm, and 56 in a subsample of surveyed control subjects).

**Discussion:**

Given increasing concerns about balancing better health outcomes with lower costs, careful examination of interventions aimed at reducing healthcare disparities attain critical importance. While analysis of the C-PNRP data is underway, the design of this patient navigation intervention will inform other patient navigation programs addressing strategies to improve prostate cancer outcomes among vulnerable populations.

## Background

Prostate cancer is the second leading cause of cancer-related deaths and the most common non-skin cancer malignancy among men in the United States, accounting for nearly one-third of all newly diagnosed cancers. An estimated 240,000 men were newly diagnosed with prostate cancer in 2011 [1]. Despite attempts to minimize gaps in health care, African American men are disproportionately burdened by prostate cancer compared to all other racial and ethnic groups in the U.S. The mortality rate for prostate cancer is an estimated 2.4 times greater among African American men than their white counterparts [2].

In 1995, an initiative that demonstrated efficacy in reducing health disparities among low-income minority women with an abnormal breast screening test in Harlem, New York, resulted in the launching of patient navigation programs nationwide [3,4]. Patient navigators are defined as advocates who interface with patients to identify and remove barriers to completing follow-up for cancer-related care [5]. In 2005, the National Cancer Institute (NCI) funded eight geographically and culturally unique sites for the Patient Navigation Research Program (PNRP); a ninth site was funded by the American Cancer Society (ACS). The PNRP aimed to improve follow-up care for undeserved individuals with abnormal screening tests for the breast, cervix, prostate, or colon/rectum [6].

The PNRP chose to navigate prostate cancer, a disease requiring complex decision-making and unequivocally diagnosed with the prostate-specific antigen (PSA) test; then, the PSA was standardized in clinical guidelines and widely utilized [7,8]. Over the past five years, increasing attention has been directed at the impact of PSA screening tests on prostate cancer mortality rates. A recent 13-year update to the cooperative Prostate, Lung, Colorectal, and Ovarian (PLCO) Cancer Screening Trial reported no significant difference in prostate cancer death rate between the screened and control groups [9]. Conversely, the 11-year update to the European Randomized Study of Screening for Prostate Cancer (ERSPC) reconfirmed that PSA-based screening reduced the rate of death from prostate cancer [10]. The continuation of conflicting results, in addition to the U.S. Preventive Services Task Force recommendation against PSA-based screening in asymptomatic men [11], underscore the need for a careful examination of patient navigation methodologies and consideration of populations likely to benefit from targeted interventions.

Evidence regarding whether an equal access health care system attenuates cancer health disparities also remains unsettled. Some studies suggest that the association between race or socioeconomic status and prostate cancer outcomes may diminish in equal access health care settings such as the Veterans Affairs (VA) system [12-14], while others demonstrate persistence of these disparities irrespective of reduced financial barriers to care [15-18]. Thus, the Chicago Patient Navigation Research Program (C-PNRP) was implemented at a VA hospital for men with an abnormal prostate screening test result. Patient navigators, working within the VA system, assisted veterans in proactively identifying barriers to keeping scheduled appointments and adhering to prostate cancer treatment modalities. Our study recruited 546 subjects with an abnormal prostate cancer screening test; data analyses are currently underway. We anticipate that our program design, planned outcome evaluations, and lessons learned presented in this paper will inform the development of future patient navigation interventions for underserved men with abnormal prostate cancer screening tests.

## Methods/Design

### Conceptual framework

We incorporated aspects of two main models to guide our navigation framework. Based on Bastani et al.’s model for intervention research, dissemination, and implementation [19] describing four levels of interventions (patient-, provider-, practice-, and policy-targeted) to improve follow-up after abnormal screening tests, the project was designed to include a multi-tiered care management team comprising a nurse practitioner, social worker, and lay health navigator and later modified to include only a social worker and lay health navigator who worked closely with clinic-based nurses. The team was formulated to address barriers present at each level and tailored navigation to the identified needs of each patient. Our navigation model was also rooted in a modified Chronic Care Model [20], representing a system rather than an individual and existing within a network framework whereby navigators assisted veterans in mitigating multifaceted barriers to obtaining follow-up care at the individual, health care system, and community levels. Our navigators were task-oriented (identifying and mitigating patient and systems barriers) and facilitative (supporting the actions of the patients).

### Study outcomes

The C-PNRP was administered through Northwestern University, and the prostate arm was fielded through a tertiary care VA hospital. The national PNRP aims included time to diagnostic resolution, time to treatment initiation, patient satisfaction, and cost-effectiveness [6]. Specific aims in Chicago included (1) increasing the proportion of patients with diagnostic evaluations among navigated men compared to controls receiving usual care; (2) for patients who receive follow-up diagnostic evaluations, improving the time between abnormal screening and diagnostic resolution compared to controls; (3) for patients with cancer, improving the time between diagnostic resolution and treatment initiation among navigated men compared to controls and; (4) identifying patient characteristics associated with diagnostic evaluation delay and patient satisfaction. Diagnostic resolution is defined as the date of the diagnostic test (either a biopsy or follow-up PSA test) that resolved the screening test into a positive cancer diagnosis or negative cancer diagnosis (either a resolution based on pathology or a PSA level/velocity determined to be within normal range). Treatment initiation is defined as the first date of treatment received for either single or combination therapy.

### Study site selection

Our study was implemented in an urban VA hospital, which provided guaranteed financial access to follow-up care after a positive prostate cancer screening test. The VA and its four community based outpatient clinics provide care for approximately 58,000 veterans in Chicago, Cook County (the county in which Chicago resides), and northwest Indiana. Nearly half of veterans in Cook County are 65 years of age or older [21], and a large proportion is African American. Previous work by our collaborators at the Chicago VA revealed that literacy may impact early-stage prostate cancer diagnosis among low-income men [22]. Moreover, among veterans with prostate cancer, older age and lower literacy skills were predictors of high PSA levels [23]. Thus, overcoming potential barriers to timely follow-up such as age, cultural beliefs, and literacy played an important role at this site.

### Needs assessment

The first year of the program was dedicated to better understanding patient and systems barriers to timely prostate cancer care at the VA. Semi-structured questionnaires were administered to two groups: key informants (medical and administrative staff) and patients who had not followed up on an abnormal screening test within 6 months. The key informant questionnaire focused on established processes for screening, diagnosis, treatment, and missed appointments. The patient questionnaire focused on screening behavior, rationales for delaying care, social support, and facilitators to care. Together, the responses helped to inform the development of the study protocol and to determine the times at which patient navigation could intersect the flow of usual care.

### Community advisory board

To inform the development, implementation, and sustainability of the navigation program, a community advisory board (CAB) was assembled. The VA advisory board met on multiple occasions throughout the project period and included leaders in their respective fields: the physician principal investigator of the project, a physician co-investigator, a former VA Chief of Staff, the director of the ACS-Illinois patient navigation services, former veteran cancer survivors, the Chief Executive Officer of a community cancer non-profit organization, and the assistant to a Congressman whose district comprises a large African American population. The members’ range of expertise in personal cancer management, clinical treatment of prostate cancer, health care systems, community outreach, and health policy contributed valuable input and feedback relative to the development of a culturally-sensitive program geared at vulnerable populations. In summary, CAB members gave voice to the underserved segments of the Chicago community.

### Study design

The study used a quasi-experimental design. Eligible patients included men 18 years of age or older with an abnormal prostate cancer screening test (PSA ≥ 4.0 ng/mL, PSA < 4.0 ng/mL with abnormal velocity, or abnormal digital rectal exam) and who, following consultation with a specialist, were recommended for biopsy. Exclusion criteria included cognitive impairment as defined by documentation of dementia in medical charts, institutionalization, previous navigation for an abnormal cancer screen or diagnosis, and a prior history of cancer except non-melanoma skin cancer that was treated within the past five years. Eligibility was reviewed via the VA’s electronic medical record system. Patients were selected to receive navigation if they met inclusion criteria and presented to the urology clinic for follow-up on a clinic day designated for the navigation intervention (n = 245). Patients who presented for follow-up on a clinic day designated for non-intervention were selected as controls (n = 245). The urology clinic was staffed with rotating resident physicians and a permanent team of attending physicians and nurses across navigation and non-navigation days.

**Figure 1:**
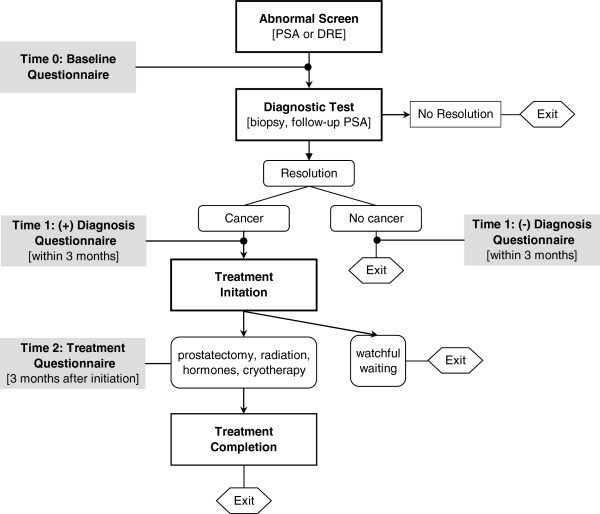
**Study flow chart.** Men were enrolled following referral for either an abnormal prostate specific antigen or digital rectal screening test and navigated through diagnostic resolution for patients without prostate cancer, treatment completion for those with prostate cancer, or end of study. Questionnaires were administered at baseline, within 3 months of diagnosis, and 3 months after treatment initiation to navigated subjects and a subsample of controls.

Patients were approached for participation in the study following a referral from a nurse practitioner or urologist. As illustrated in Figure 1, patient navigators facilitated assistance through diagnostic resolution for patients without prostate cancer, completion of primary therapy for patients with prostate cancer, or end of study. The control group received usual care and comprised a concurrent records-based sample. A subsample of control subjects who completed a diagnostic evaluation (n = 56) were prospectively enrolled for the questionnaire portion of the study only. Written informed consent and authorization were obtained from the navigated and prospective control patients, and a waiver of authorization was granted for the records-based control arm. All study procedures were approved by the Northwestern University Institutional Review Board (IRB), Jesse Brown VA Medical Center Research & Development Committee, and the Collaborative IRB of the University of Illinois at Chicago.

### Data collection and survey administration

Clinical data elements such as visit dates, test results, and baseline comorbid conditions [24] were abstracted from the VA’s computerized electronic medical record system (CPRS) by trained research personnel in accordance with the data dictionary developed by the PNRP [6]. CPRS allowed researchers to access primary care records from VA community-based outpatient clinics as well as treatment records from other VA tertiary care centers. Data were initially recorded on case report forms and subsequently entered into electronic databases. As part of quality control and assurance measures, a subset of chart audits were independently performed by teams of research personnel (N.N., P.B., T.H., and E.R.) to ensure uniformity in data collection. Any missing data or discrepancies were documented and adjudicated by clinical investigators (M.S. and J.M.). The program and data manager resolved all responses in the final dataset.

Psychosocial assessments were administered to participants in person or by telephone at baseline, following diagnosis, and if applicable, after treatment initiation (Figure 1). Table 1 illustrates questionnaire measures and specific administration time points. For navigated participants, data on demographics, health literacy [25], perceived stress [26], social support [27], locus of control [28], health care system distrust [29], self-efficacy [30], impact of events [31], patient satisfaction with care [32], and patient satisfaction with navigation [33] scales were collected. For the subsample of prospective control participants, trained research assistants collected data on demographics, self-efficacy, impact of events, and satisfaction with care at diagnosis and treatment to contribute to the multi-center PNRP analysis. 

**Table 1:** Psychosocial questionnaire measures and administration times

**Baseline/outcome measure**	**Questionnaire**	**Survey administration times**			
		**Baseline**	**Time 1**		**Time 2**
		**(after referral for abnormal screen)**	**(within 3 months of*negative*diagnosis)**	**(within 3 months of*positive*diagnosis)**	**(3 months after treatment initiation)**
Health literacy	Rapid Estimate of Adult Literacy in Medicine [25]	X			
Comorbid conditions that may alter risk of mortality	Charlson Comorbidity Index [24]	X			
Anxiety and stress	Perceived Stress Scale [26]	X			
Perceived functional support	Medical Outcomes Study-Social Support Survey [27]	X			
Predisposition for fatalistic attitudes directed at health	Wallston Multi-Dimensional Locus of Control [28]	X			X
Distrust towards health care system	Health Care System Distrust [29]	X	X		X
Patient engagement in own health care	*Communication and Attitudinal Self-Efficacy - General [30]		X		
Patient engagement in own cancer care	*Communication and Attitudinal Self-Efficacy - Cancer [30]				X
Subjective distress related to a specific event	*Impact of Events [31]		X	X	X
Satisfaction with cancer-related care	*Patient Satisfaction with Care [32]		X	X	X
Satisfaction with patient navigation services	Patient Satisfaction with Navigator [33]		X	X	X

*Administered to a subsample of prospective control participants for national PNRP analysis.

In addition to clinical and psychosocial data, patient navigators recorded each activity they performed for or on behalf of the patient on tracking logs. Data elements included encounter date, encounter type (telephone, in-person, interaction with third party), length of encounter, barrier type, actions taken to resolve barrier, and length of time to complete each action.

### Patient navigators

#### Recruitment and characteristics of navigators

The navigation team consisted of two full-time navigators: a social worker with an advanced degree and a college-educated lay health worker. A nurse practitioner was also included in the original navigation framework; however, because the nurse practitioner’s navigation role duplicated clinical services present within the VA, such as cancer treatment education, we modified the team to include a lead social worker and lay patient navigator who regularly collaborated with clinical providers at the VA. The navigator team consisted of white and African American navigators, mirroring the cultural composition of the VA. Additionally, because the study population was exclusively male veterans with gender-specific care needs, the project actively sought male patient navigators.

#### Training of patient navigators

Patient navigators were trained on a national, state, and local level. Navigators attended annual training conferences sponsored by the NCI and the ACS, which focused on exposure to various patient navigator types, development of resource tools for patients, and enhancement of communication and collaboration skills [34]. At the state level, the lead social worker navigator participated in monthly patient navigation-focused training sessions facilitated through the ACS-Illinois. Locally, study investigators trained all navigators on the consent process, research surveys, and cultural and communication barriers. Further, discussion of challenging cases, effective navigation strategies, and clinical terminology were incorporated into weekly meetings. Navigators also completed human subject research training and participated in relevant VA human subjects, patient education, and electronic medical record training sessions at the clinic site as needed. Navigators were evaluated at the clinic site on a quarterly basis and supervised by the project manager and two ACS representatives specializing in patient navigation services.

### Patient navigator intervention

#### Navigation activities

The navigation team was formally credentialed by the VA and directly interfaced with patients both in person and by telephone. On the day of enrollment, navigators assessed for barriers in person in the urology clinic. To address the barriers, navigators completed activities, such as interacting with providers and the healthcare system on patients’ behalf, addressing psychosocial issues, performing appointment reminder calls, linking patients with resources, coordinating transportation, and facilitating patient education and responses to clinical questions. Moreover, since navigators were based in the VA with access to updated electronic medical records, they were able to monitor patients’ progress in real-time and meet with them face-to face at clinic visits, particularly treatment appointments in order to provide social support. Our navigators worked with medical and non-medical clinic staff to resolve emerging issues, and if unable to do so with their assistance, connected patients with VA and community organizations. Though each patient navigator had his individual caseload, the social worker navigator provided consultation to the lay navigator on issues that required specialized expertise.

Navigation intensity (total time spent with or on behalf of a patient) varied on a case-by-case basis and was determined predominantly by the patient’s existing social support network, barriers identified, and selection of treatments that required repeat visits, such as radiation therapy. At minimum, each patient received an initial assessment of barriers and standardized appointment reminders. On average, navigators spent twice as much time with patients who had cancer compared with those without cancer.

#### Appointment reminder system

Navigators completed systematic appointment reminders to prevent missed clinic visits and aided patients in canceling or rescheduling appointments when necessary to avoid delays in diagnostic resolution. Navigators also made initial reminder telephone calls to each patient one month prior to his scheduled prostate needle biopsy and utilized these calls to review procedure-related preparatory instructions, since lack of adherence to these instructions often resulted in a rescheduled appointment. For patients who could not be directly reached at their valid primary telephone number, navigators left a voicemail appointment reminder upon repeat contact. A second appointment reminder was made ten days prior to the scheduled biopsy to ensure that patients remained adherent to preparatory instructions. If the patient, through previous interaction with their navigator, expressed difficulty remembering appointment times, the navigator performed a third reminder call prior to the biopsy date. Patients diagnosed with prostate cancer received additional reminder calls throughout treatment as needed.

### Data analysis plan

National outcome measures will be evaluated by the Patient Navigation Research Program [6,35,36]. Table 2 presents the patient characteristics of the study sample for our local analysis (n = 490). The majority was greater than 65 years of age (53%), African American (68%), and referred to our program based on an abnormal PSA test (84%); over one-third were diagnosed with prostate cancer. Plans to analyze our local outcomes are described below. 

**Table 2:** Patient characteristics, abnormal screen group (n = 490)

	**All subjects (n = 490)**
	***N (%)***
**Age**	
< 65 years	261 (53%)
> = 65 years	229 (47%)
Mean	65.1
Median	63.5
Range	42-92
**Race/ethnicity**	
African American	332 (68%)
White	105 (21%)
Other	27 (6%)
Not reported	26 (5%)
**Screening eligibility**	
PSA test (> = 4.0 ng/mL)	414 (84%)
Abnormal PSA velocity	39 (8%)
Digital rectal exam	37 (8%)
**Baseline PSA**	
< 4 ng/mL*	37 (8%)
4 ng/mL - 10 ng/mL	318 (65%)
> 10 ng/mL	98 (20%)
Not collected (Screen = DRE)	37 (8%)
**Cancer diagnosis**	
No cancer or resolved with repeat PSA	244 (50%)
Cancer	170 (35%)
Unresolved	76 (16%)

Abbreviations: PSA, prostate specific antigen; ng/mL, Nanogram per milliliter; DRE, Digital rectal exam.

*Values may not sum to all subjects who enrolled on abnormal PSA velocity due to rounded PSA value.

#### Power calculation

Based on our group’s previous prostate cancer research at the VA, we estimated that the mean time from abnormal screen to diagnostic resolution (Time 1) in the control group would be 103 days and the mean time from cancer diagnosis to treatment initiation (Time 2) in cancer patients would be 128 days. The initial power calculation indicated that for Time 1, 300 patients per group would have 80% power to detect a mean Time 1 of 86 days in the navigated group, and that 120 cancer patients per group would have 80% power to detect a mean Time 2 of 90 days in the navigated group. Since only 245 rather than 300 patients were enrolled per group for Time 1, the actual effect size for Time 1 that was detectable with 80% power was 103 days versus 84 days. Since only 85 rather than 120 patients were enrolled per group for Time 2, the actual effect size for Time 2 that was detectable with 80% power was 128 days versus 82 days. Two-tailed tests and a Type I error rate of 5% were assumed.

#### Primary outcomes

To evaluate our primary outcome measures, we will perform unadjusted and adjusted time-to-event analyses for time to diagnostic resolution and time to treatment initiation. Since patients were followed for one year, events that occurred within 365 days from abnormal screen for Time 1 or 365 days from diagnosis for Time 2 will be considered events in the statistical analysis. For unadjusted analyses, we will calculate Kaplan-Meier curves, use them to determine median times within groups, and compare curves between groups using the log-rank test. For adjusted analyses, we will use proportional hazards regression. Covariates will include age and race for Time 1, and age, race, and Gleason score for Time 2. In addition to using a p-value testing whether the hazard ratio (HR) was 1.0 or not, we will use adjusted hazard ratios (HR) and 95% confidence intervals.

#### Exploratory analyses

For secondary outcomes, exploratory analysis will be conducted to evaluate patient characteristics that may be associated with delay of or non-compliance with diagnostic resolution in the intervention arm. Descriptive statistics will be obtained for each variable. Demographic characteristics and baseline scores on each survey instrument will be uniformly entered into one model for analysis, and multivariate linear regression models will be used to evaluate the relationship between potential predictor variables (literacy, stress, social support, distrust, etc.) and the dependent variable: days between abnormal prostate screening test and diagnostic resolution. In addition to multivariate linear regression models, multiple logistic regression models will be used to explore the relationship between demographic or psychosocial variables and overall adherence to screening follow-up, patient satisfaction, and navigation intensity.

## Discussion

Patient navigation programs have been primarily undertaken within the breast cancer setting [5,37]. Because prostate cancer differs in disease progression and quality of life implications, however, the investigation of the efficacy of patient navigation within the prostate cancer continuum is warranted. Weinrich et al. previously reported that the use of a prostate cancer education program combined with a navigator resulted in increased prostate cancer screening rates among African American men [38]. Yet, to date, no studies have been published on the effect of patient navigation on compliance with prostate cancer diagnosis or treatment among men with an abnormal prostate screening test. Our study is the first to evaluate these outcomes.

Given equivocal evidence presented in the PLCO and ERSPC studies on the efficacy of prostate cancer screening in reducing mortality [39,40], attention needs to be directed at intervention entry points (screening, cancer diagnosis, treatment), particularly for prostate patient navigation interventions. It is well settled that an elevated PSA can represent several medical conditions beyond prostate cancer, including prostatitis and benign prostatic hypertrophy. PSA can also be artificially and transiently elevated by recent digital rectal examination, prostate needle biopsy, and recent sexual activity. Therefore, the limitations of an abnormal PSA test alone must be taken into consideration when implementing a navigation intervention. Our study adequately addressed this concern, as it focused on men whose prostate cancer screening results, history, and clinical presentation to the urologist reflected a high probability of prostate cancer, thus resulting in a referral for biopsy.

In addition, our study was uniquely implemented at a VA hospital, an equal-access setting with a range of preventive and treatment services. Prior to implementation of the PNRP, most studies reporting on efficacy of navigation on screening, diagnosis, or treatment had been conducted in the community clinic setting [5,37]. Despite reduced financial barriers to entry, however, a study examining over thirteen thousand veterans with an abnormal prostate cancer screening test result found that one-third received incomplete follow-up [41]. Our study site involved rotating resident physicians from two large academic research hospitals, at times introducing disconnect in provider-patient relationships, which may impact resolution rate [42,43]. Our patient navigators filled this gap by building rapport and trust through the tenure of the patient’s cancer care continuum to ensure that abnormal screens reached resolution.

While the methods we outlined in this study were compatible with our clinic site, they may not be generalizable across non-VA facilities, facilities with multiple genders, different geographic regions, and non-urban settings. Given centralized care at the VA, we chose a hospital-based framework, whereas other PNRP programs extended into patients’ homes and communities to best meet the needs of their targeted populations [44]. Additionally, the average age of men in our study was 65, mandating clinical considerations more specific to the elderly and the weighing of risks and benefits of the more aggressive therapies usually reserved for and selected by younger patients, particularly within the VA [45]. While caution relative to overtreatment resonates across all age groups in the literature, some studies raise the issue of under treatment of cancer among the elderly and support risk-stratified approaches to prostate cancer care management [46,47]. Against this backdrop, a large component of our patient navigation intervention focused on facilitating patient education and connecting patients with resources to guide them through the treatment decision-making process.

Throughout the implementation of the intervention, we learned many valuable lessons that may benefit emerging navigation programs. First, as previously described, in response to repetition between the roles of our initial nurse practitioner navigator and clinic-based nurses, we adapted our model to include a social worker and lay health navigator who worked closely with the VA nurses to facilitate clinical needs. Programs planning navigation team composition should consider utilizing existing providers with the requisite knowledge. This approach supports a smooth clinical research transition and facilitates timely study initiation. Second, our project was originally designed to prospectively enroll navigated and control participants at two demographically similar VA outpatient clinics. Due to a hospital merger prior to accrual, we adapted our design to include a concurrent records-based control group drawn from the same VA clinic as the navigated patients. Future navigation studies should similarly be prepared to adapt quickly to unanticipated clinic site changes. Finally, since our navigators lacked access to the VA appointment scheduling system, they focused on patient adherence to scheduled visits to prevent delayed follow-up. Thus, intervention approaches that similarly comprise proactive measures—envisioning and actuating issues that could introduce delay—rather than reactively resolving patient-identified barriers can be invaluable in reaching targeted navigation goals.

## Conclusion

Racial/ethnic disparities in prostate cancer outcomes have been well documented, and patient navigation represents one strategy that has proven effective in improving timeliness of diagnostic resolution in community health centers and breast cancer settings. The goal of our patient navigation study was not to alter prostate cancer screening practices at the VA hospital; rather, we sought to work within the existing systems framework to provide a more patient-centered and disease-specific approach to ensuring patient adherence with prostate cancer care. We focused on navigating low-income individuals within the VA with an abnormal finding consistent with prostate cancer, which arguably might not address the potential navigational needs of similarly situated men outside the VA system. Further research that utilizes novel methodologies and is directed at examining navigation interventions for men with prostate cancer who lack VA support is needed.

## Competing interests

The authors declare that they have no competing interests.

## Authors’ contributions

MS, JM, NN, and PB contributed to the needs assessment, design of the study, and development of the research protocol. NN coordinated the study and drafted the manuscript, and JM, AS and XD participated in critical manuscript revision. PB supervised the navigation team, TL and ER assisted with data management, and GP assisted with data collection and manuscript revision. AR and DL performed the statistical analyses, and AR, MS, and NN were involved in the interpretation of the data. All authors have read and approved the final manuscript.

## Pre-publication history

The pre-publication history for this paper can be accessed here:

http://www.biomedcentral.com/1472-6963/12/340/prepub
